# Improvement in racial disparities in years of life lost in the USA since 1990

**DOI:** 10.1371/journal.pone.0194308

**Published:** 2018-04-25

**Authors:** Jeanine M. Buchanich, Shannon M. Doerfler, Michael F. Lann, Gary M. Marsh, Donald S. Burke

**Affiliations:** 1 Department of Biostatistics, Graduate School of Public Health, University of Pittsburgh, Pittsburgh, PA, United States of America; 2 Graduate School of Public Health, University of Pittsburgh, Pittsburgh, PA, United States of America; New York City Department of Health and Mental Hygiene, UNITED STATES

## Abstract

**Objective:**

To examine changes in cause-specific Years of Life Lost (YLL) by age, race, and sex group in the USA from 1990 to 2014.

**Methods:**

60 million death reports from the National Center for Health Statistics (NCHS) were categorized by age group, sex, race, and cause of death. YLL were calculated using age-specific life expectancies. Age groups were: infants <1, children 1–19, adults 20–64, and older adults 65+.

**Results:**

Blacks have historically experienced more years of life lost than whites or other racial groups in the USA. In the year 1990 the YLL per 100,000 population was 21,103 for blacks, 14,160 for whites, and 7,417 for others. Between 1990 and 2014 overall YLL in the USA improved by 10%, but with marked variations in the rate of change across age, race, and sex groups. Blacks (all ages, both sexes) showed substantial improvement with a 28% reduction in YLL, compared to whites (all ages, both sexes) who showed a 4% reduction. Among blacks, improvements were seen in all age groups: reductions of 43%, 48%, 28%, and 25% among infants, children, adults, and older adults, respectively. Among whites, reductions of 33%, 44%, and 18% were seen in infants, children, and older adults, but there was a 6% increase in YLL among white adults. YLL increased by 18% in white adult females and declined 1% in white adult males. American Indian/Alaska Native women also had worsening in YLL, with an 8% increase. Asian Pacific Islanders consistently had the lowest YLL across all ages. Whites had a higher proportion of YLL due to overdose; blacks had a higher proportion due to homicide at younger ages and to heart disease at older ages.

**Conclusions:**

Race-based disparities in YLL in the USA since 1990 have narrowed considerably, largely as a result of improvements among blacks compared to whites. Adult white and American Indian / Alaskan Native females have experienced worsening YLL, while white males have experienced essentially no change. If recent trajectories continue, adult black/white disparities in YLL will continue to narrow.

## Introduction

Racial disparities in health have been evident in the US for decades[1–3]. These have been attributed to inequity in social determinants of health, such as income and education[3–5], environmental hazards, such as proximity to road traffic and increased air pollution[6], differences in behavioral risk factors, such as smoking, and access to quality health care[6–8]. Improvements in black-white differences occurred in the 1970s and early 1980s, but increased again in the late 1980s due primarily to lower improvements in heart disease mortality rates and HIV-related deaths among blacks[9].

Recent examinations of mortality patterns in the US have focused on changes over time by race and sex. Acciai et al.[10] (2015) have attributed the higher life expectancy among Asian and Pacific Islanders in the US to Asians outliving whites regardless of cause of death (i.e., Asians have a higher average age of death for almost all causes of death). Case and Deaton[11] (2015) found increasing mortality rates among young and middle age non-Hispanic white adults which they attributed to increasing rates of accidental overdose, suicide, and liver disease. National Center for Health Statistics (NCHS) data indicate that death rates from Alzheimer’s disease, liver disease and cirrhosis, and suicide rose between 2014 and 2015[12]. More recently, Shiels et al.[13] examined changes in mortality rates by race, sex, age group, and cause of death in the US from 1999 to 2014. These examinations focused on changes in life expectancy and mortality, but not changes in years of life lost.

Years of life lost (YLL) is a measure of premature death calculated by summing the number of years each death occurs before some “target” age to which all people could be expected to live, such as life expectancy. This measure gives more weight to deaths occurring among young people. YLL provides local regional, or national public health officials with the ability to quickly examine differential rates and identify appropriate targets for intervention(s) in their ability to prevent premature death [14]. Cause-specific YLL may give stakeholders the additional information needed to most accurately and effectively target scarce resources to address gaps and eliminate disparities [15]. Examinations of cause-specific YLL help identify targets for cause-specific policy and practice changes.

Studies [16–18] have examined differences in YLL by sex; however, to date no study has analyzed cause-specific YLL in the US by age-race-sex groups. The purpose of this study was to examine changing patterns in cause-specific YLL in the US since 1990 by age, race, and sex.

## Methods

Death data and population counts were extracted from the Mortality Information and Research Analytics (MOIRA) system (formerly known as the Mortality and Population Data System (MPDS [19]), which is a repository and retrieval system for detailed mortality data obtained from the National Center for Health Statistics (NCHS) and the US Census. The database contains death counts and populations for the entire US and at the state and county level by race, sex, and age group.

The MOIRA was developed and, since 1980, has been maintained by the University of Pittsburgh Graduate School of Public Health Department of Biostatistics[19]. It contains the underlying cause of death code (using International Classification of Diseases (ICD) codes) for all persons who died in the US between 1950 and 2014. It is limited to deaths from malignant neoplasms for the 1950–1961 time period. The MOIRA is updated annually and currently contains information on more than 117 million US deaths.

For this study, death records (N = 59,777,652 deaths) were used that included information on race, sex, age group at death (<1, 1–4, 5–9, 10–14, 15–19, 20, 24, 25–34, 35–44,45–54, 55–64, 65–74, 75–84, 85+), year of death (1990–2014), and specific cause of death (ICD code). For 1990–1998, race was classified as white, black, and other per NCHS guidelines. These data did not have ethnicity information. From 1999–2014, more detailed race information was available: white, black, Asian-Pacific Islander (API), and American Indian–Alaska Native (AI/AN). Information on ethnicity (Hispanic/non-Hispanic) was also available for 1999–2014. Death certificate information on race and ethnicity is recorded as it was reported by the decedent’s informant. MOIRA data were used to generate cause-specific YLL by race-sex group. The MOIRA cause of death categories (Appendix A) were collapsed into 10 larger categories to allow more meaningful examinations of YLL (Appendix B). External causes of death were divided into four categories: non-drug overdose accidents (transport, falls, drowning, suffocation), suicide, homicide, and accidental drug poisoning (drug overdose). Infectious diseases, diseases of the heart, and cancers were aggregated into one group each. All other causes were separated into either: organ diseases (blood, endocrine, respiratory, digestive, skin, musculoskeletal, and genitourinary systems); pregnancy, perinatal and congenital conditions; or all others (primarily mental/behavioral, nervous system, and “signs and symptoms not otherwise classified”). Comparability ratios were not applied to account for the change from the 9^th^ to the 10^th^ revision ICD because they were not available for our aggregated causes of death. Analyses of counts of death by year by group did not indicate problems with these groups across revisions (data not shown).

YLL was calculated for four age groups: <1, 1–19, 20–64, and 65+. Years of life lost among those <1, 1–19, 20–64 and 65+ were calculated by multiplying the number of deaths within each of 13 age groups by the average remaining life expectancy (in years) of that age group using the US life expectancy data from the Global Burden of Disease Study 2013[20] for each year (Appendix C). The total YLL formula, for all age groups, is given below:

$$YLL=\sum_{i=1}^{m}d_{i}*{le}_{i}$$

where *i* indexes the age group, and *m* represents the number of age groups. The number of deaths and average life expectancy (in years) at age *i* are denoted by *d*_*i*_ and *le*_*i*_, respectively.

Cause-specific YLL was calculated by summing the years of life lost within each cause of death category by age group. In the same way, race-sex specific YLL was calculated by age group within the respective race-sex groups. Analyses for 1990–2014 were by age group for white, black, and other races. Specific race information on deaths comprising the ‘other’ race group (API and AI/AN) and for ethnicity (Hispanic/non-Hispanic) was available from 1999 to 2014. For that time period, we examined cause-specific and age-specific YLL for AI/AN and API groups, and for Hispanic/non-Hispanic groups. Deaths, YLL count, and YLL rate for each race-sex group by age group and cause of death group are found in Appendix D.

## Results

**Fig 1:**
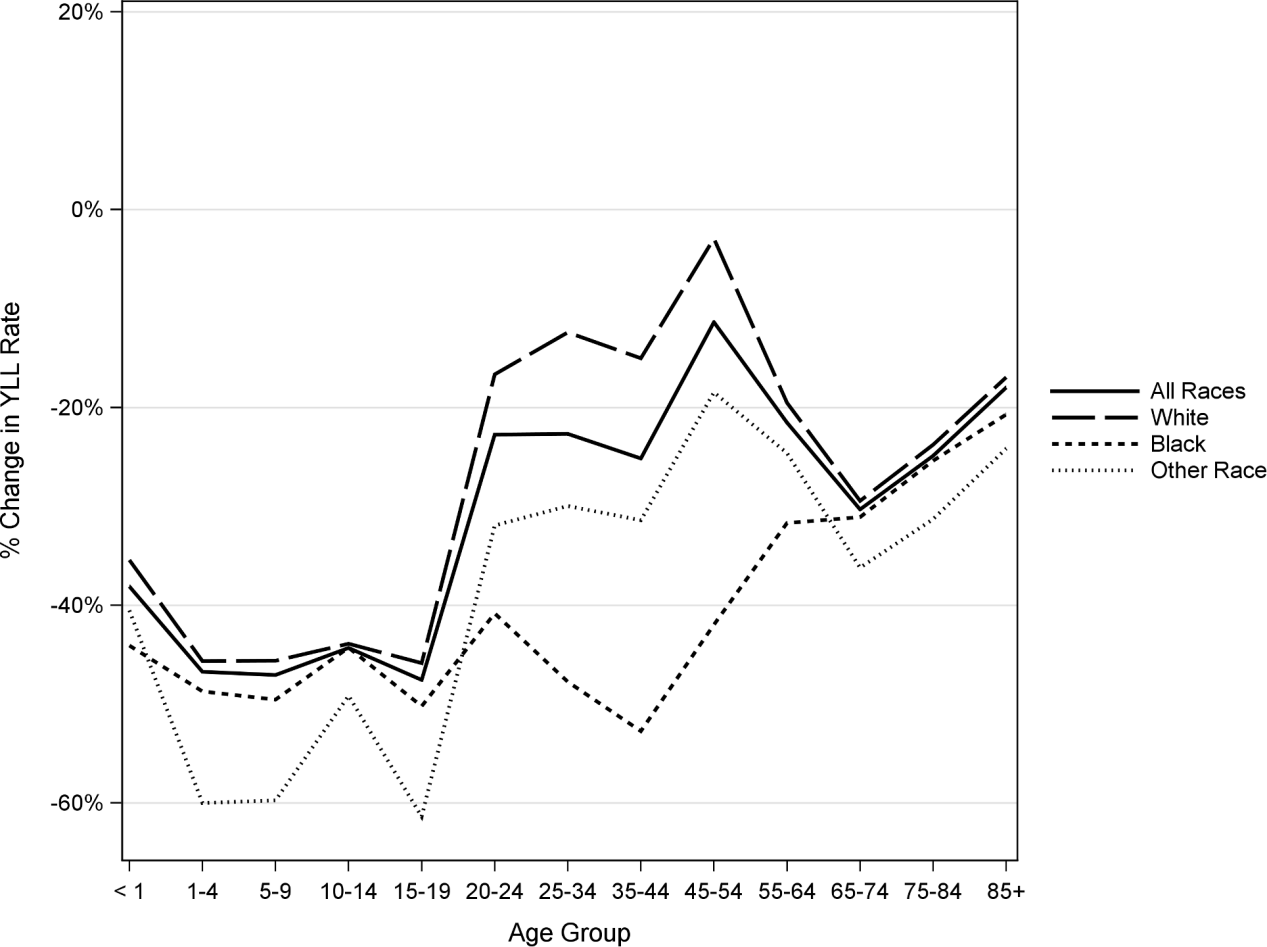
Percent change in YLL rates from 1990 to 2014 for US males by race and age.

Males of all races have seen improvement in YLL rate since 1990 for all age groups (Fig 1). Males of other races had the greatest improvement among children (ages 1–19), while black males had the greatest improvement among adults (ages 20–64) with a reduction of 53% in 35–44 year olds. White adult males have had the least amount of improvement and there are few differences in the rate of improvement among older adults males.

**Fig 2:**
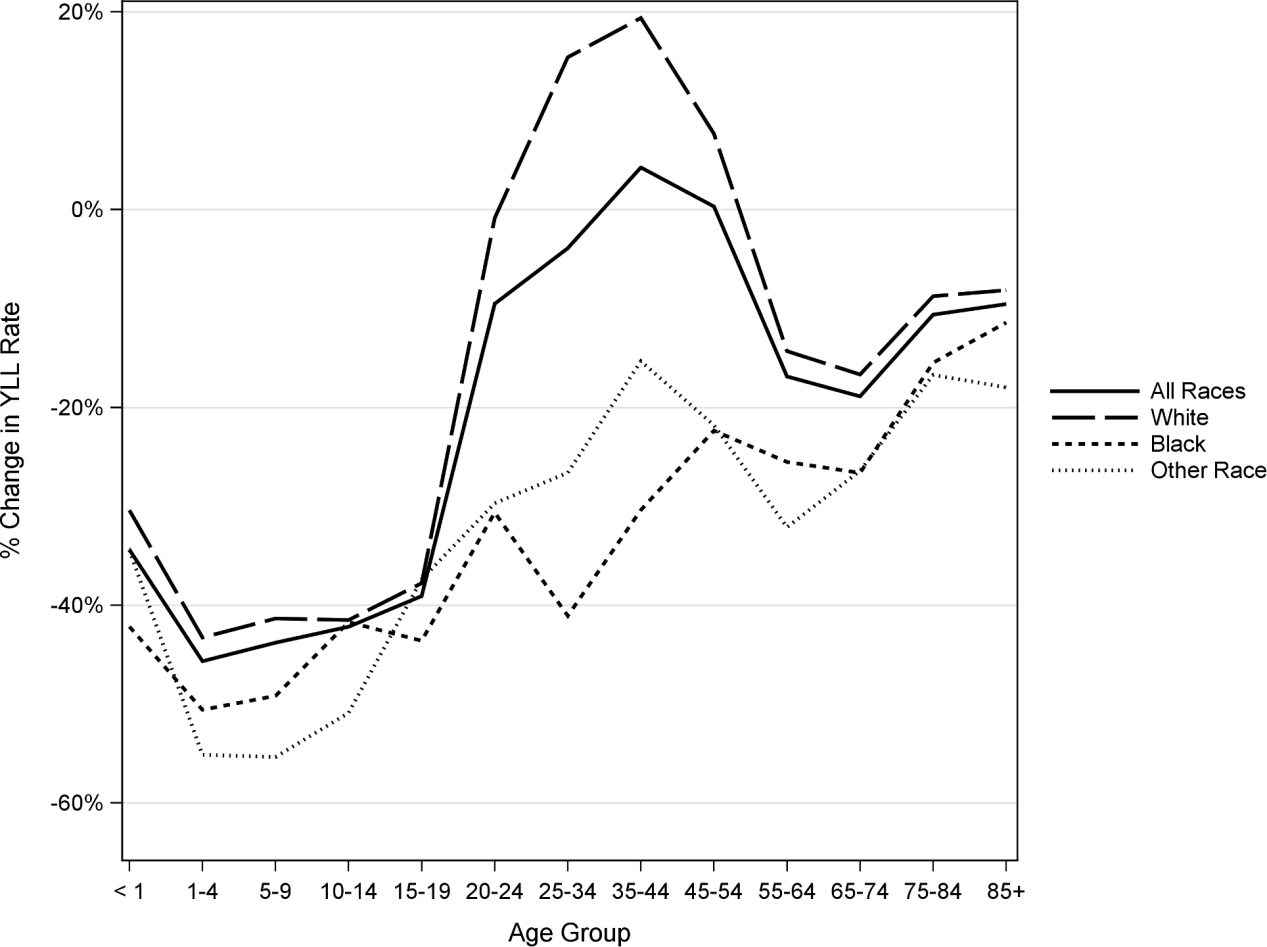
Percent change in YLL rates from 1990 to 2014 for US females by race and age.

Results are similar for the females (Fig 2). Other races had the greatest improvements among most age groups of children, while black females had the largest decreases for ages 25–44. However, white female YLL has increased since 1990 in each of the age groups 25–54, by 15%, 19%, and 8% respectively. White females have also seen less improvement for ages 55+ than have black females and females of other races.

Change in YLL rates by race-sex group and age group is shown in Table 1. Also shown is the percent of racial disparity for blacks and other races (as compared to whites) for YLL by sex and age group in 1990 and in 2014. In 1990 among all ages, the disparities by race were -57% and 46%, respectively for black males and males of other races compared to white males. In 2014, those disparities were -33% and 52%. For females, the disparities by race were -40% for black females compared to white females and 50% for females of other races compared to white females. In 2014, these disparities were -10% and 55%. The largest decrease in percent disparity between 1990 and 2014 for black males and black females was among ages 20 to 64, from 52% to 26% and from 48% to 27%., respectively. Other age groups had more modest decreases in percent disparity.

**Table 1:** YLL rates per 100,000 population, percent disparity, and percent change (in YLL per 100,000) from 1990 to 2014 by race, sex, and age group.

	All Ages					<1					1 to 19					20 to 65					65+				
	1990	% disp.^1^	2014	% disp.^1^	% change^2^	1990	% disp.^1^	2014	% disp.^1^	% change^2^	1990	% disp.^1^	2014	% disp.^1^	% change^2^	1990	% disp.^1^	2014	% disp.^1^	% change^2^	1990	% disp.^1^	2014	% disp.^1^	% change^2^
White Male	17222	—	15809	—	-8	67565	—	43622	—	-35	3560	—	1948	—	-45	14228	—	14100	—	-1	60144	—	44432	—	-26
Black Male	27100	-57	18164	-15%	-33	159272	-136	89048	-104	-44	6103	-71	3112	-60	-49	29366	-106	19072	-35	-35	78681	-31	55676	-25	-29
Other Male	9316	46	7556	52%	-19	55108	18	32770	25	-41	3373	5	1349	31	-60	8545	40	7114	50	-17	38178	37	25521	43	-33
White Female	11225	—	11315	—	1	52025	—	36206	—	-30	1964	—	1160	—	-41	7067	—	8327	—	18	37515	—	33024	—	-12
Black Female	15720	-40	12436	-10%	-21	130859	-152	75666	-109	-42	2978	-52	1566	-35	-47	13500	-91	11387	-37	-16	47705	-27	37342	-13	-22
Other Female	5585	50	5092	55%	-9	43004	17	28199	22	-34	1756	11	896	23	-49	4570	35	3983	52	-13	22850	39	18524	44	-19
All White	14160	—	13541	—	-4	59994	—	39997	—	-33	2784	—	1563	—	-44	10632	—	11225	—	6	46628	—	38095	—	-18
All Black	21103	-49	15182	-12%	-28	145215	-142	82499	-106	-43	4557	-64	2352	-50	-48	20881	-96	15052	-34	-28	59599	-28	44691	-17	-25
All Other	7417	48	6283	54%	-15	49185	18	30535	24	-38	2583	7	1126	28	-56	6494	39	5486	51	-16	29657	36	21575	43	-27
All Male	18092	—	15520	—	-14	81646	—	50528	—	-38	3935	—	2094	—	-47	15672	—	14227	—	-9	61122	—	44416	—	-27
All Female	11577	36	11000	29%	-5	64523	21	42335	16	-34	2111	46	1207	42	-43	7759	50	8412	41	8	38108	38	32705	26	-14
All	14753	—	13225	—	-10	73281	—	46522	—	-37	3045	—	1660	—	-45	11664	—	11301	—	-3	47359	—	37859	—	-20

^1 –^Calculated as percent difference in 1990 YLL or 2014 YLL using first row of group as baseline (e.g.,((17222–27100)/17222)*100 = - 36%)

^2-^ Calculated as percent difference between 1990 YLL and 2014 YLL within a row

Since 1990 for all age groups and races, males have had larger improvements in YLL than have females (14% reduction in males compared to a 5% reduction in females). Males of other races age 1–19 had the greatest improvement of any group examined, with a 60% decrease in YLL in the last 25 years. White females age 20–64 have had the worst improvement of any group examined, with an 18% increase in YLL from 1990 to 2014. The ratio of YLL for blacks to whites was the lowest for ages 65+ in 2014 (YLL_Black/YLL_White = 117) and highest in 2014 for infants (YLL_Black/YLL_White = 206). The ratio of YLL for blacks to whites had the biggest improvement for ages 20 to 64 from 1990 to 2014, although this is in part due to increasing YLL among white females in this age group (an increase of 26% among white females age 25–54).

The Supplementary Figs show changes from 1990 to 2014 in YLL by age group and race for the specific cause of death groups (S1–S5 Figs). For adults, ages 20–64, males of other races have the lowest YLL and the lowest burden from each cause group (Panel A in S1 Fig). Blacks have the highest proportion of YLL due to homicide in young ages and heart disease in older ages. Whites have the largest proportion of YLL due to overdose. Findings are similar in adult females (Panel B in S1 Fig), with white females having the largest burden due to overdose and black females having the largest burden due to heart disease.

In the older adults (ages 65+), YLL due to heart disease is highest for black males, especially in ages 65–74 (Panel A in S2 Fig). The patterns in females for YLL in ages 65+ are very similar to those in males (Panel B in S2 Fig). YLL due to other causes has increased since 1990 for males and females ages 65+ (data not shown); this category is comprised mainly of dementia and Alzheimer’s disease in these age groups.

Black male children (ages 1–19) have higher YLL compared to whites and other races; the difference is due mostly to higher YLL from homicide (Panel A in S3 Fig). White and black males have higher YLL due to accidents than males of other races. Also evident among white males is the burden of YLL due to overdose and suicide beginning around age 10. YLL in female children has also dropped since 1990 (Panel B in S3 Fig). YLL for ages 15–19 is very similar in black and white females, although both are higher than females of other races. Black females also have a greater burden of YLL due to homicide, although not as much as in black males (Panel B in S3 Fig). Once again, YLL in white females begins to be affected by overdose and suicide at age 10.

Examinations for age <1 by race for males and females found that perinatal causes are not surprisingly the leading cause of YLL in 2014 in this age group (data not shown). Black males have the largest burden of YLL due to perinatal causes, and also due organ diseases, infections, and accidents. Black females have also seen the greatest reduction among the race groups examined (42%—Panel B in S1 Fig). Similar to males, YLL in black females is most often due to perinatal conditions, but also to “other” causes of death. In infants, that category is mainly comprised of deaths coded to “Symptoms, signs, and abnormal clinical and laboratory findings, not elsewhere classified” (ICD-10 codes R00-R99).

Panel A in S4 Fig shows, for 1999 to 2014, YLL decreased slightly among almost all ages between 20 to 64 years old for both AI/AN and API males. YLL increased slightly for API males ages 20 to 24. YLL rates in 2014 for AI/AN are similar to those in white males (Panel A in S1 Fig), although AI/AN males have higher YLL due to accidents at younger ages. API males have the lowest YLL of any race group examined across all ages, with the largest component of YLL due to suicides in younger age groups and to cancers in older age groups. YLL among AI/AN females has increased since 1999 in ages 35 to 54 (Panel B in S4 Fig), but has decreased for all ages among API females. YLL among AI/AN females ages 20 to 64 is also similar to that of white females (Panel B in S1 Fig). AI/AN females have a higher burden of YLL due to accidents in younger ages, similar YLL due to overdose as found in white females, and higher burdens due to infections and organ diseases in older ages. API females have the lowest YLL of any race group examined for all ages.

A comparison by ethnicity (Hispanic/non-Hispanic) is shown in Panels A and B in S5 Fig. Among males (Panel A in S5 Fig), non-Hispanics had greater decreases in YLL across all ages 20–64 than Hispanics. Non-Hispanic males had decreases in YLL for all age groups. Hispanic males age 25–34 had a 16% increase in YLL from 1999 to 2014. Hispanic males had greater YLL due to overdose and suicide YLL in younger adults (20–35) and in cancer among older adults (45–64) compared to non-Hispanic males. Compared to 1999, Hispanic females had YLL increases of 13%, 5%, and 12% among 25–34, 35–44, and 45–54 year olds, respectively, in 2014 (Panel B in S5 Fig). Non-Hispanic females had YLL decreases in the same time period for each age group among ages 20–64. Hispanic females had higher YLL from every cause of death category examined compared to non-Hispanic females.

## Discussion

This study is the first to examine cause-specific patterns of YLL by age, race, and sex groups. Race-based disparities in YLL have decreased over the last 25 years. Compared to whites and other races, black males and females had the largest reductions in YLL in all age groups, except among 1–19 year olds. Males and females of other races had the lowest YLL at every age. This was due to lower YLL among API males and females for all age groups. Two thirds of the deaths among other races in 1999 were among API; by 2014, that proportion had increased to 70%. Whites had a higher proportion due to overdose at all ages. Blacks had a higher proportion of YLL due to homicide at younger ages and to heart disease at older ages. We found that homicide mortality rates among black males declined in the 1990s for all age groups 20–64, but have remained steady since 1999 (data not shown). For ages 65+, there were fewer variations by race as heart disease, cancers, and other causes (dementia and Alzheimer’s disease) accounted for the most YLL.

The increase in YLL in white women ages 20 to 64, and specifically a 26% increase among 25–54 year olds since 1990 is particularly concerning. Case and Deaton[11] found increasing mortality rates among young and middle age non-Hispanic white adults due primarily to increasing rates of accidental overdose. Shiels et al.[13] found increases in mortality rates among white females 25–39 and 40–49 and among white males age 25–39, attributed primarily to increases in accident mortality. In this study, YLL among white males age 20 to 64 remained essentially unchanged (1% decrease). We did find a disproportionate effect of overdose in young and middle aged white males and females.

These findings correspond in some ways to recent examinations of mortality rates in the US. Acciai[10] found lower mortality rates in Asians (in this study, part of “other” races for analyses covering 1990 to 2014) for all causes across all ages, which coincides with our findings. After using age-incidence methodology to decompose the age and incidence components of the mortality rates, they attributed this to Asians having a higher average age at death for almost all causes of death. The age component from heart disease contributed almost 2 years toward life expectancy and cancer added another 1.4 years. Other conditions also contributed to the 6.9 year life expectancy gap between Asians and whites found in the paper. However, the authors could not attribute this difference to any particular lifestyle, genetic, or environmental factors. Shiels et al.[13] found that mortality rates for Asian and Pacific Islanders dropped for all age groups examined in males and females, with no or only very slight increases in accident mortality. We found that Asians in this study had lower YLL for all causes of death for every age group examined. We did find, however, that accident YLL increased among Asian females <1 year old by 11%, older adults females (75+ years old) by 31% and elderly males (85+ years old) by 5% from 1999 to 2014.

A 2013 study published by the Burden of Disease collaborators found the highest counts of age-adjusted years of potential life lost (YPLL), using life expectancy at birth, from ischemic heart disease, lung cancer, stroke, chronic obstructive lung disease (COPD), and road injury[16]. Ma et al. [17] examined rates of change from 1969 to 2013 in years of potential life lost at age 75 (YPLL75) for six leading causes of death[18]. They found decreases in diabetes, cancer, unintentional injuries, heart disease and stroke, but no change in COPD. Our age-specific findings placed less emphasis on stroke and COPD, but we did find high proportion of YLL due to ischemic heart disease among all race-sex groups in older ages, and a high proportion due to lung cancer, especially in white females. Among younger ages, we found a large burden due to overdose in whites, and to homicides in blacks.

There is some evidence that mortality, life expectancy, and YLL disparities are related more to place than to race[21]. A recent analysis by Chetty et al.[22] (2016) found that life expectancy varied by geography and income, with those in the highest income percentiles experiencing the longest life expectancies. They also found that these differences increased over time, but that there was considerable geographic variation across states. Ezzati et al.[23] found groups of counties with statistically significant decreases in life expectancy, compared to the national average, for groups of counties predominantly throughout Appalachia and the South. A next step in these analyses is to examine geographic-specific patterns by race, sex, and age group, especially with regard to cause-specific disparities. Areas (counties or states) in which YLL is low for a particular race-sex group, or cause of death, could provide important information about interventions, policies, or lifestyles that have been successful in reducing YLL.

Like all studies relying on death certificate data, these data are subject to incomplete information recorded as the cause of death. Race on death certificates is reported by the decedent’s informant, typically the next of kin. Some evaluations have found that death certificate race is not classified the same as informant-based race of surveys, leading to misclassification and biased estimates[24, 25]. Race for blacks and whites is subject to very low misclassification, but race for AI/AN especially showed inconsistency[26]. We did not attempt to reclassify race from that reported on the death certificate. Additionally, detailed race and ethnicity were only available for mortality beginning in 1999. This restriction limited our ability to analyze patterns of YLL for API, AI/AN, and Hispanics for the entire 1990–2014 time period.

Cause of death coding relies on the text written by the coroner or medical examiner and, as such, is only as complete and accurate as reported by that person. We did not make any effort to reclassify coded causes of death to other categories which may not be appropriate as the underlying cause, as has been done for some causes of death in the Global Burden of Disease study[27, 28]. We instead chose to evaluate all cause of death codes exactly as they have been reported to the NCHS, and do not anticipate that this would have a major impact on our findings. A further examination of differences in underlying cause of death coding assignment by reporting jurisdictions within the US may be warranted. Such an examination could elucidate areas in which causes of death are more often being recorded to less meaningful underlying cause of death categories, sometimes referred to as “garbage codes” [19, 26] and could provide important information to stakeholders for actionable change.

We analyzed data by age group, rather than by single ages due to limitations in the data. A comparison of the US population by age group in 1990 and 2014 (data not shown) indicated that there are proportionately more people over the age of 45 in 2014. However, we chose not to show age-adjusted rates because we wanted to maintain the age group specific comparisons which revealed important differences by cause of death group. We chose to use overall, and not race or sex specific, life expectancy in our calculations. We felt that it was important that each specific race-sex group be compared to the same standard and not have, for example, a lower life expectancy standard for black males than for white males.

## Conclusions

This study provides important information of the differences in cause-specific YLL by age, race, and sex. Changes since 1990 indicate progress in YLL among black males and females. While YLL rates among blacks are still higher than those found in whites, dramatic improvements have been made. On the contrary, young and middle age white, Hispanic and AI/AN females have had worsening YLL over the last 25 years, and white males have seen no improvement. API males and females have the lowest YLL for all ages. Further evaluations should consider whether any areas in the US have been more successful in improving YLL for race-sex groups or for certain causes of death.

## Supporting information

S1 Fig A. 2014 YLL by cause of death group for males by race, ages 20–64, with comparison to and percent change from 1990 baseline. B. 2014 YLL by cause of death group for females by race, ages 20–64, with comparison to and percent change from 1990 baseline.
(DOCX)

S2 Fig A. 2014 YLL by cause of death group for males by race, ages 65+, with comparison to and percent change from 1990 baseline. B. 2014 YLL by cause of death group for females by race, ages 65+, with comparison to and percent change from 1990 baseline.
(DOCX)

S3 Fig A. 2014 YLL by cause of death group for males by race, ages 1–19, with comparison to and percent change from 1990 baseline. B. 2014 YLL by cause of death group for females by race, ages 1–19, with comparison to and percent change from 1990 baseline.
(DOCX)

S4 Fig A. 2014 YLL by cause of death group for males by race for AI/AN and API, ages 20–64, with comparison to and percent change from 1990 baseline. B. 2014 YLL by cause of death group for females by race for AI/AN and API, ages 20–64, with comparison to and percent change from 1990 baseline.
(DOCX)

S5 Fig A. 2014 YLL by cause of death group for Hispanic and non-Hispanic males, ages 20–64, with comparison to and percent change from 1990 baseline. B. 2014 YLL by cause of death group for Hispanic and non-Hispanic females, ages 20–64, with comparison to and percent change from 1990 baseline.
(DOCX)
