# Supplementary figures and images for: Improvement in racial disparities in years of life lost in the USA since 1990
Source: PLoS One. 2018 Apr 25;13(4):e0194308. doi: 10.1371/journal.pone.0194308 (PMC5918944; doi:10.1371/journal.pone.0194308)

S1a Fig.
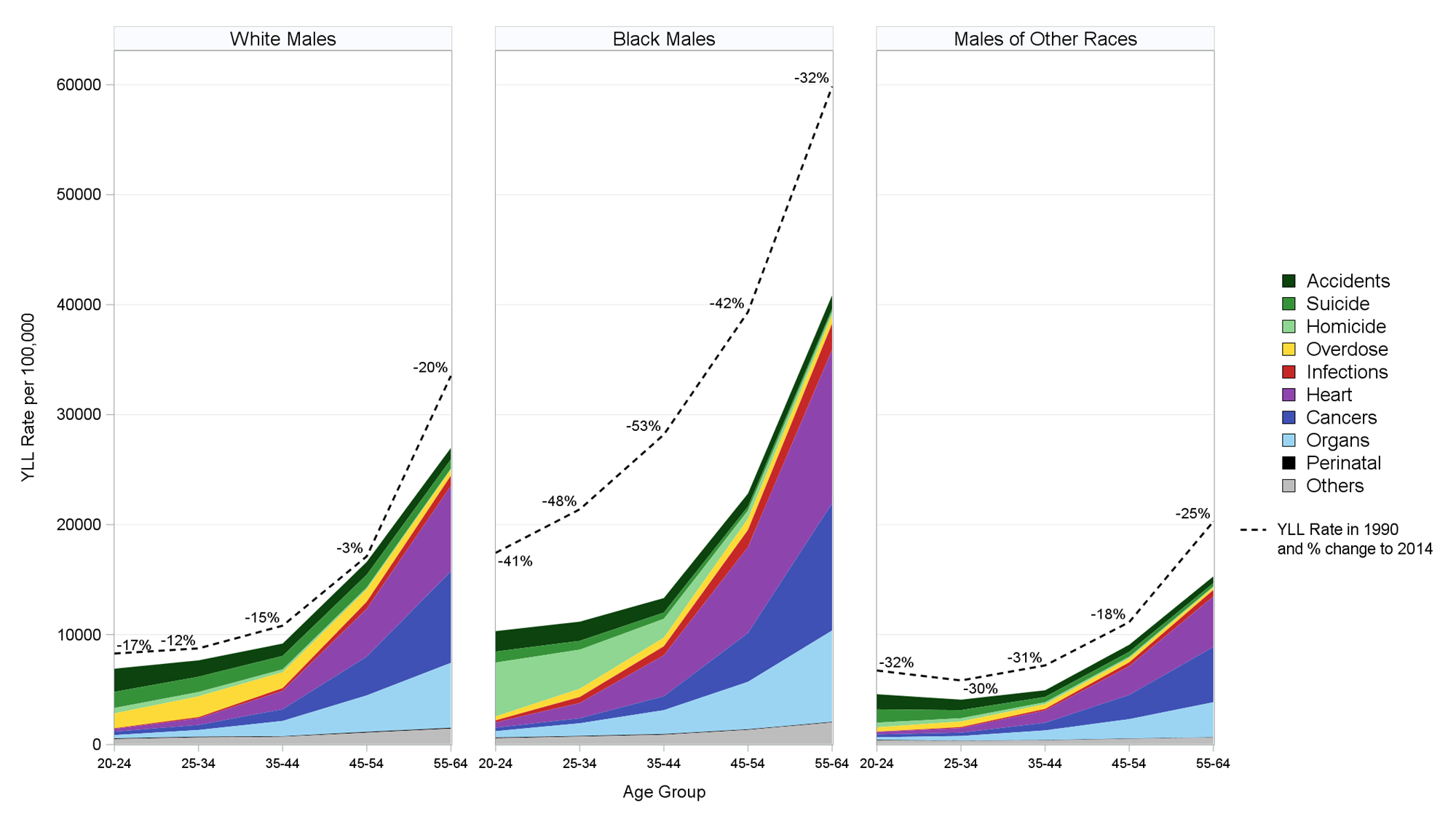
S1b Fig.
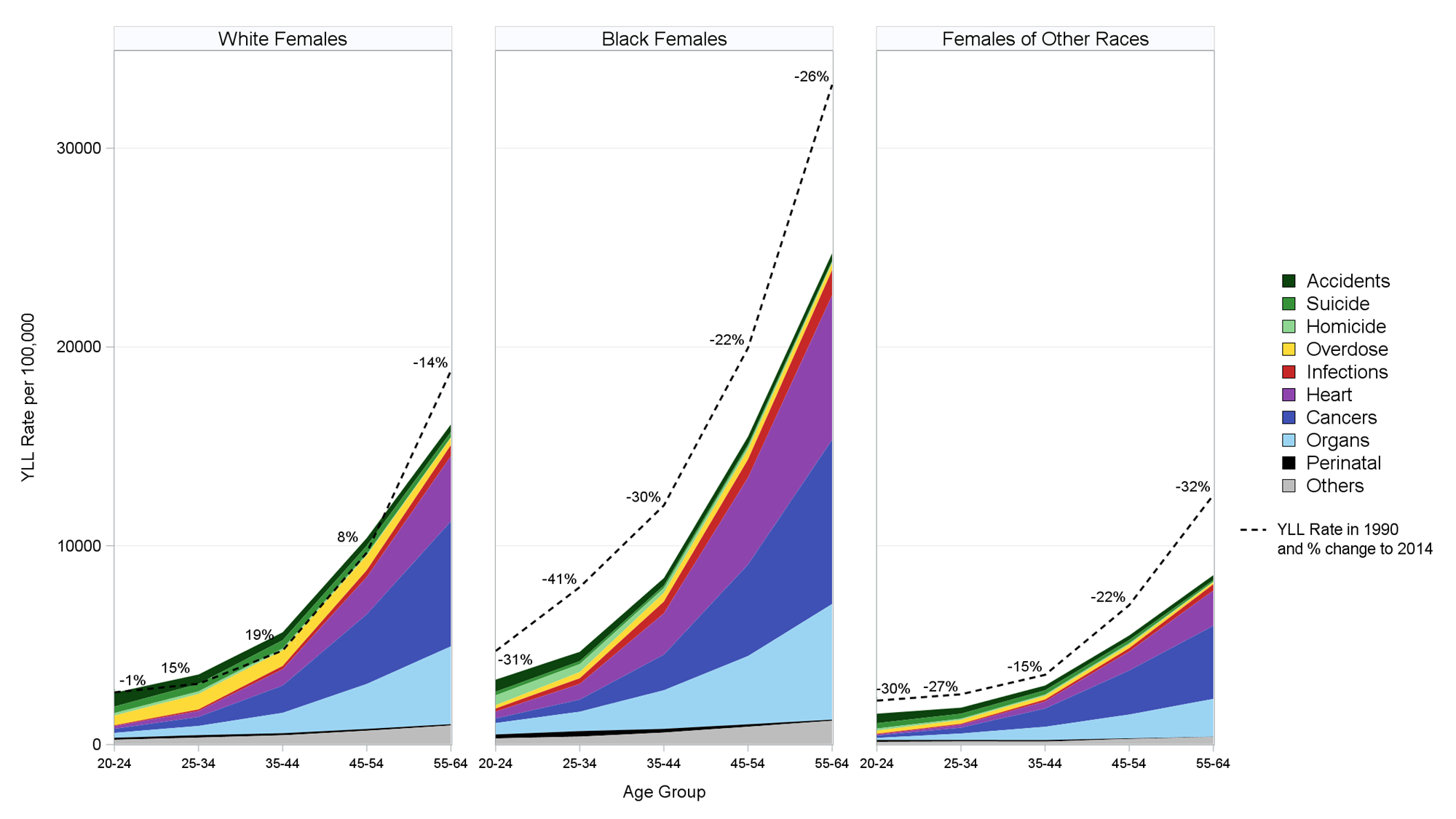

Supplement: S1 Fig — A. 2014 YLL by cause of death group for males by race, ages 20–64, with comparison to and percent change from 1990 baseline. B. 2014 YLL by cause of death group for females by race, ages 20–64, with comparison to and percent change from 1990 baseline. (DOCX) [file pone.0194308.s001.docx]

S2a Fig.


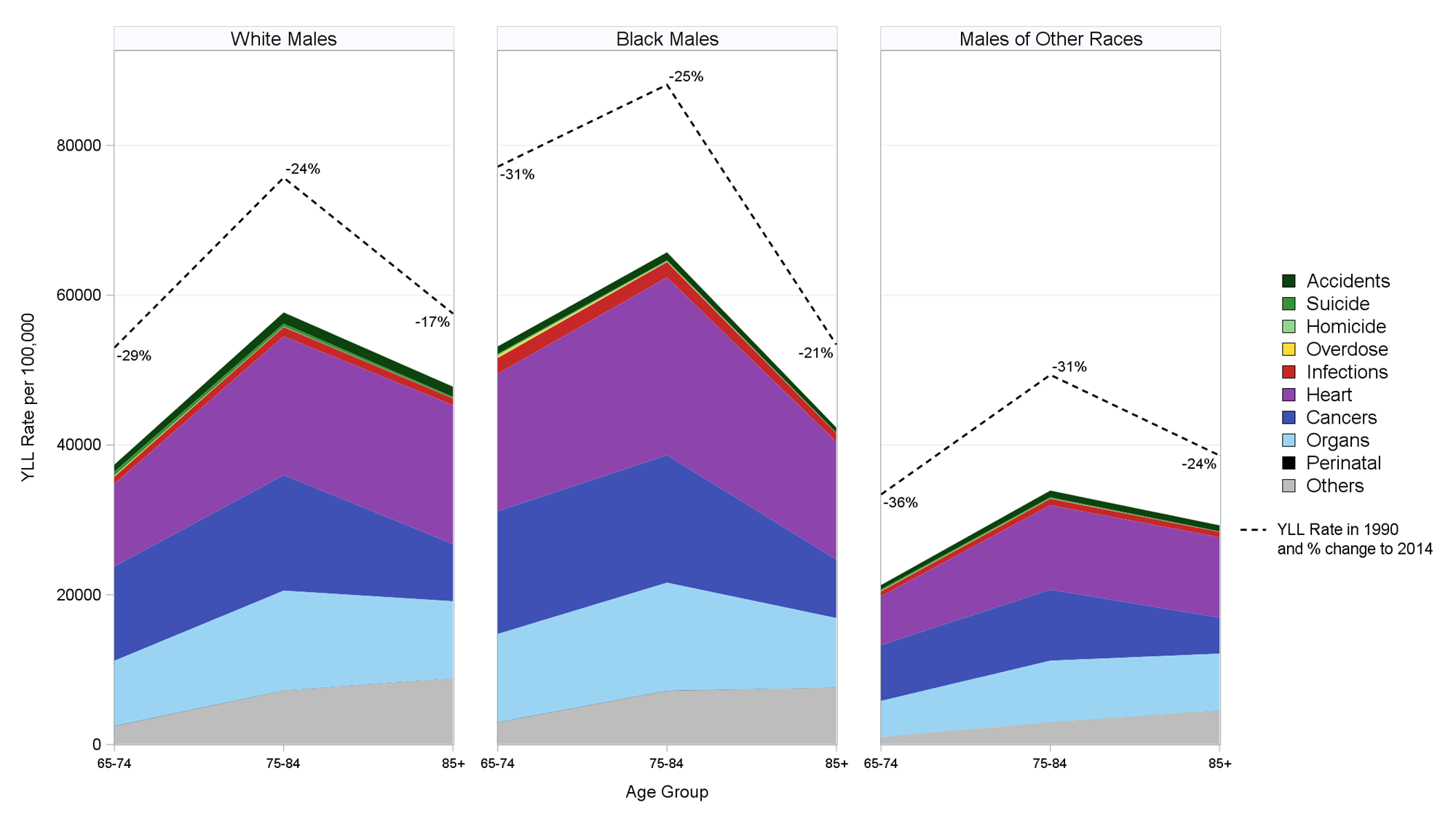


S2b Fig.


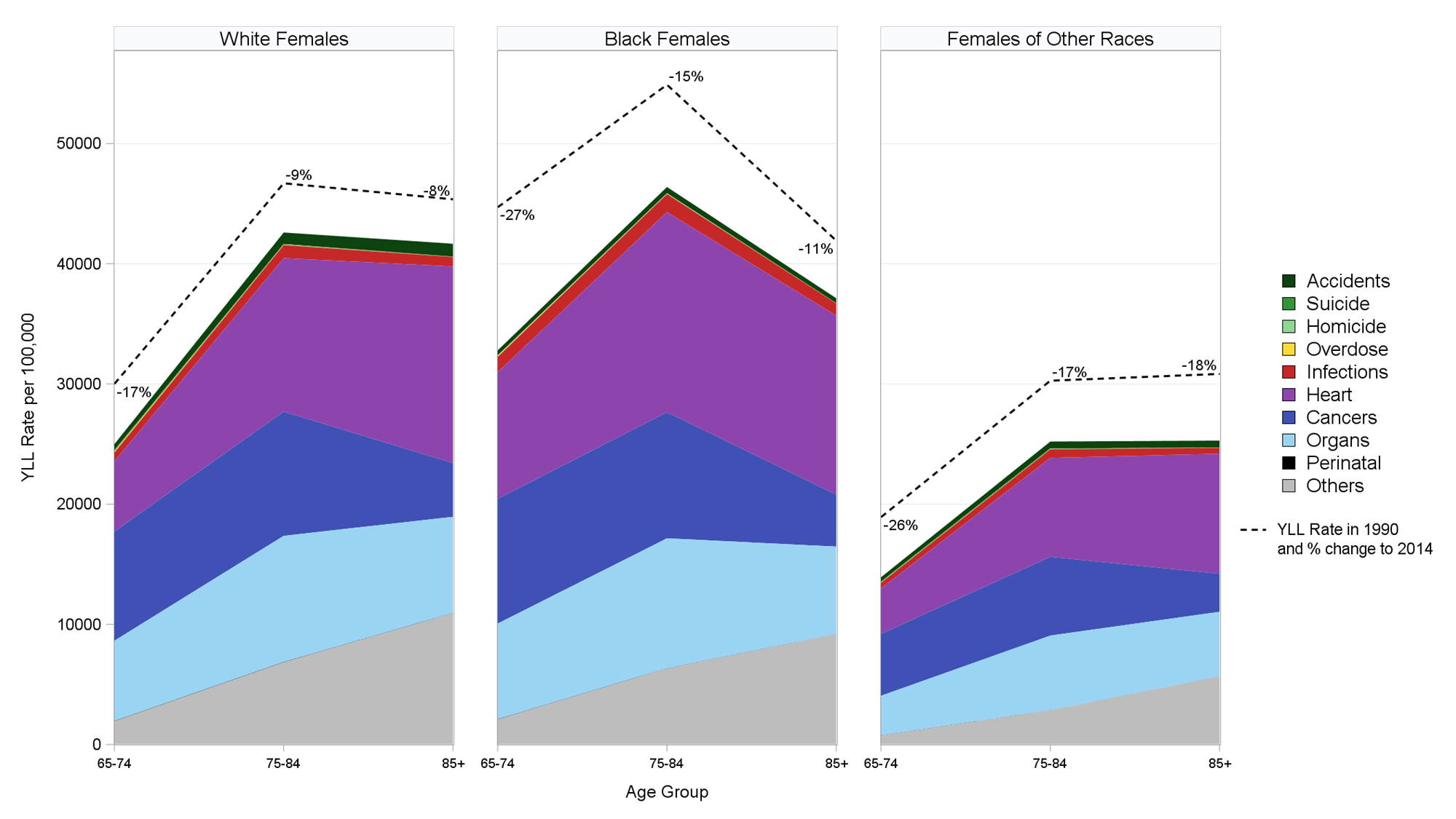

Supplement: S2 Fig — A. 2014 YLL by cause of death group for males by race, ages 65+, with comparison to and percent change from 1990 baseline. B. 2014 YLL by cause of death group for females by race, ages 65+, with comparison to and percent change from 1990 baseline. (DOCX) [file pone.0194308.s002.docx]

S3a Fig.


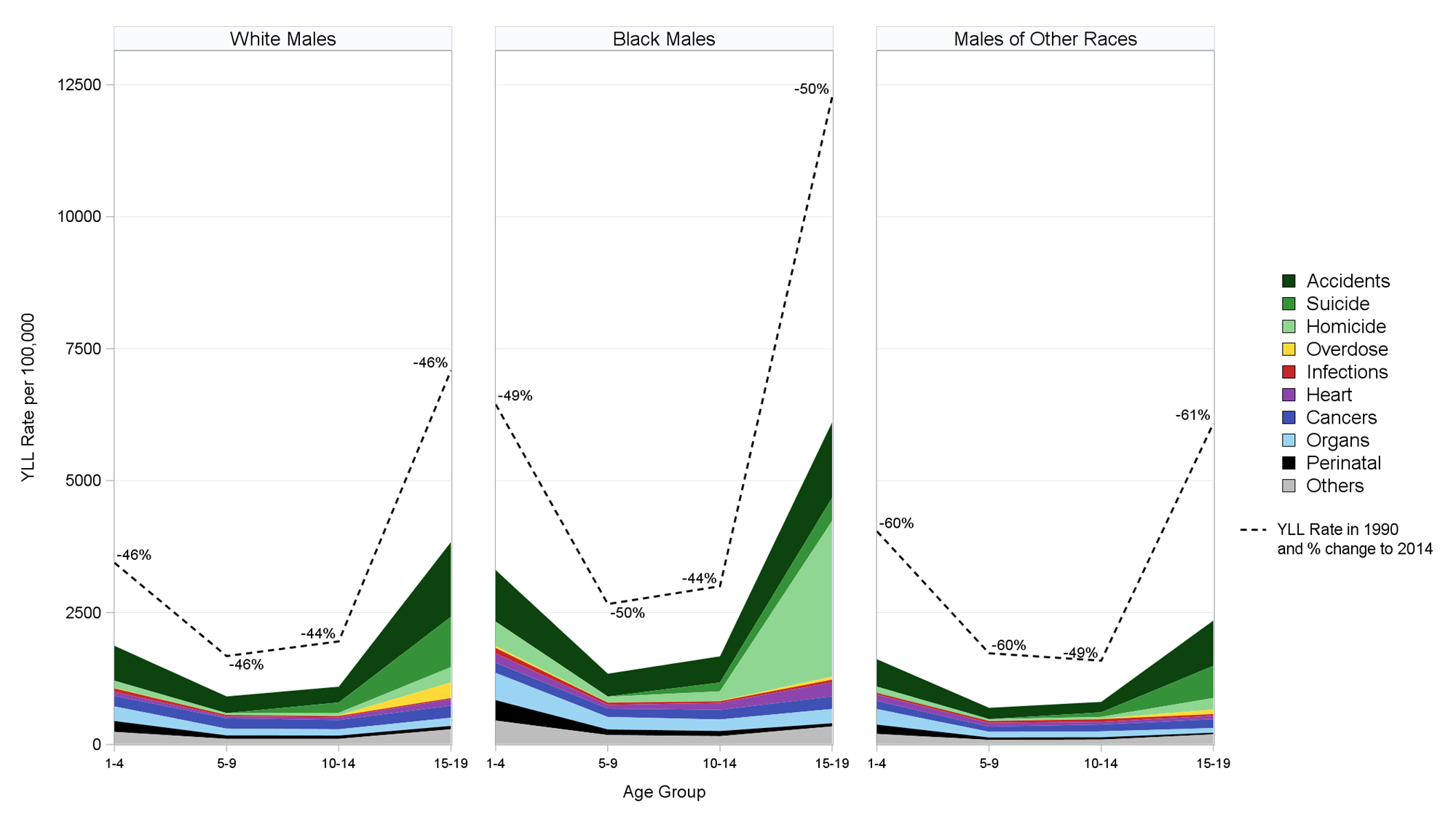


S3b Fig.


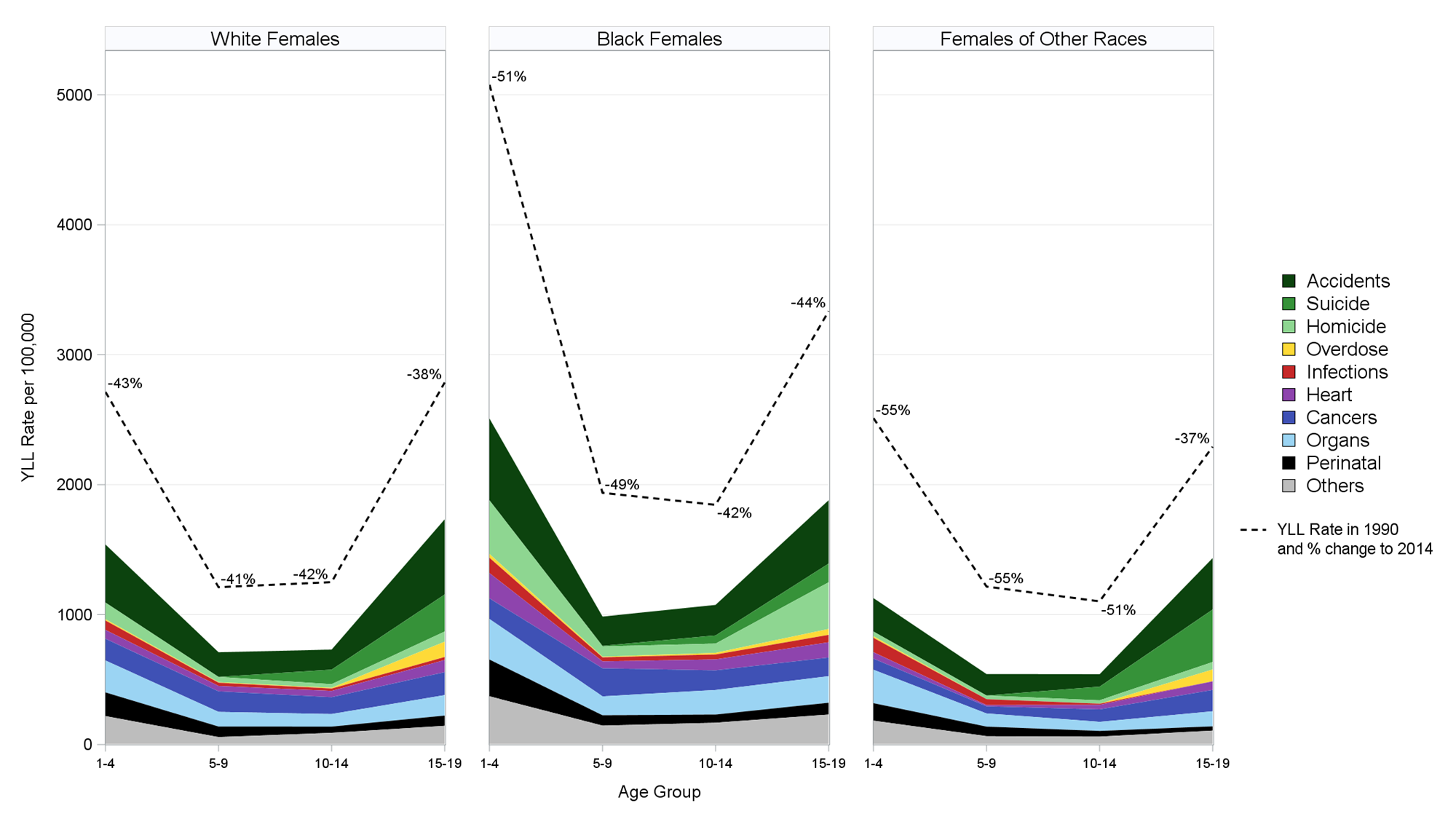

Supplement: S3 Fig — A. 2014 YLL by cause of death group for males by race, ages 1–19, with comparison to and percent change from 1990 baseline. B. 2014 YLL by cause of death group for females by race, ages 1–19, with comparison to and percent change from 1990 baseline. (DOCX) [file pone.0194308.s003.docx]

S4a Fig.


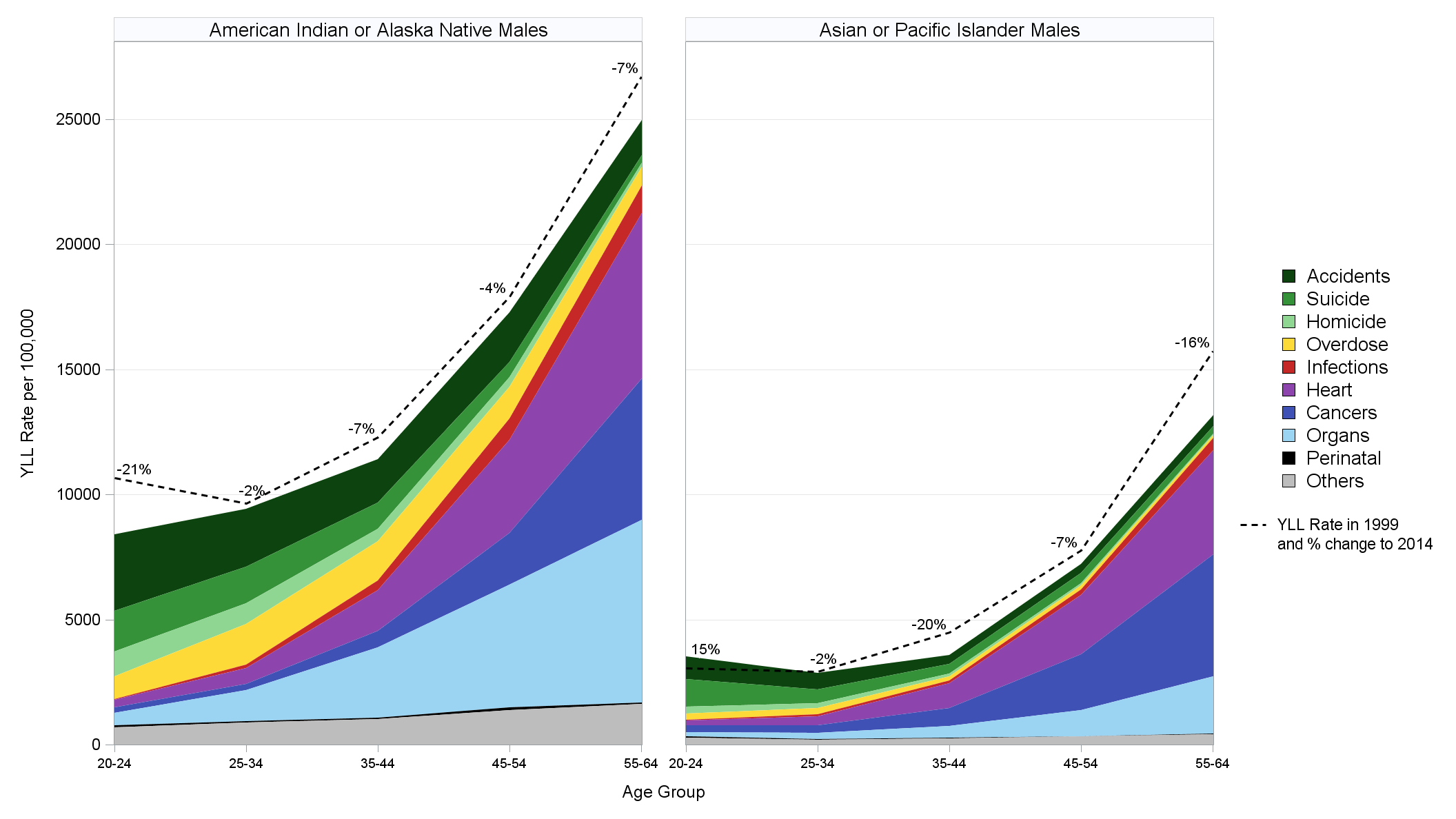


S4b Fig.

**
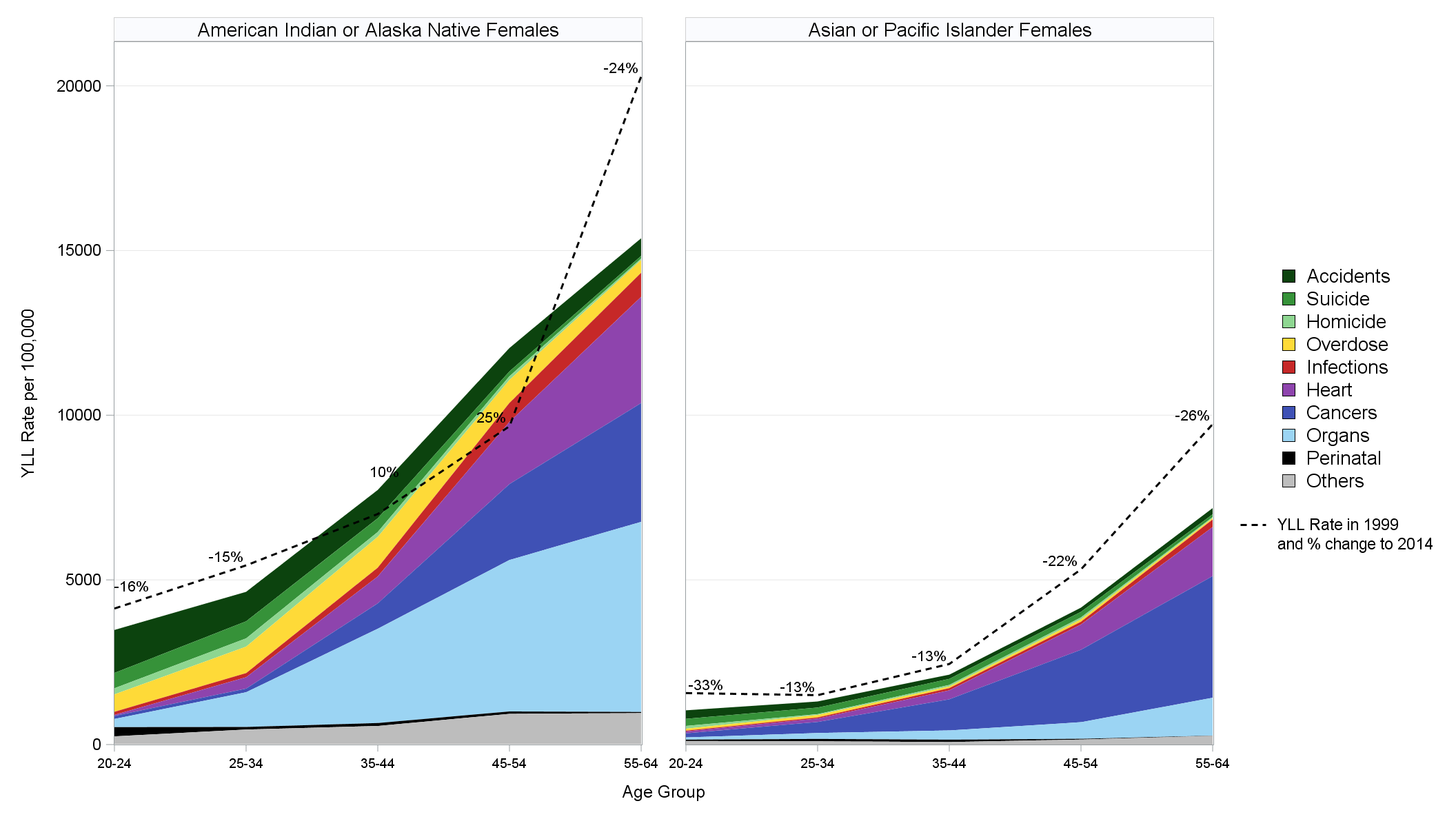
**

Supplement: S4 Fig — A. 2014 YLL by cause of death group for males by race for AI/AN and API, ages 20–64, with comparison to and percent change from 1990 baseline. B. 2014 YLL by cause of death group for females by race for AI/AN and API, ages 20–64, with comparison to and percent change from 1990 baseline. (DOCX) [file pone.0194308.s004.docx]

S5a Fig.


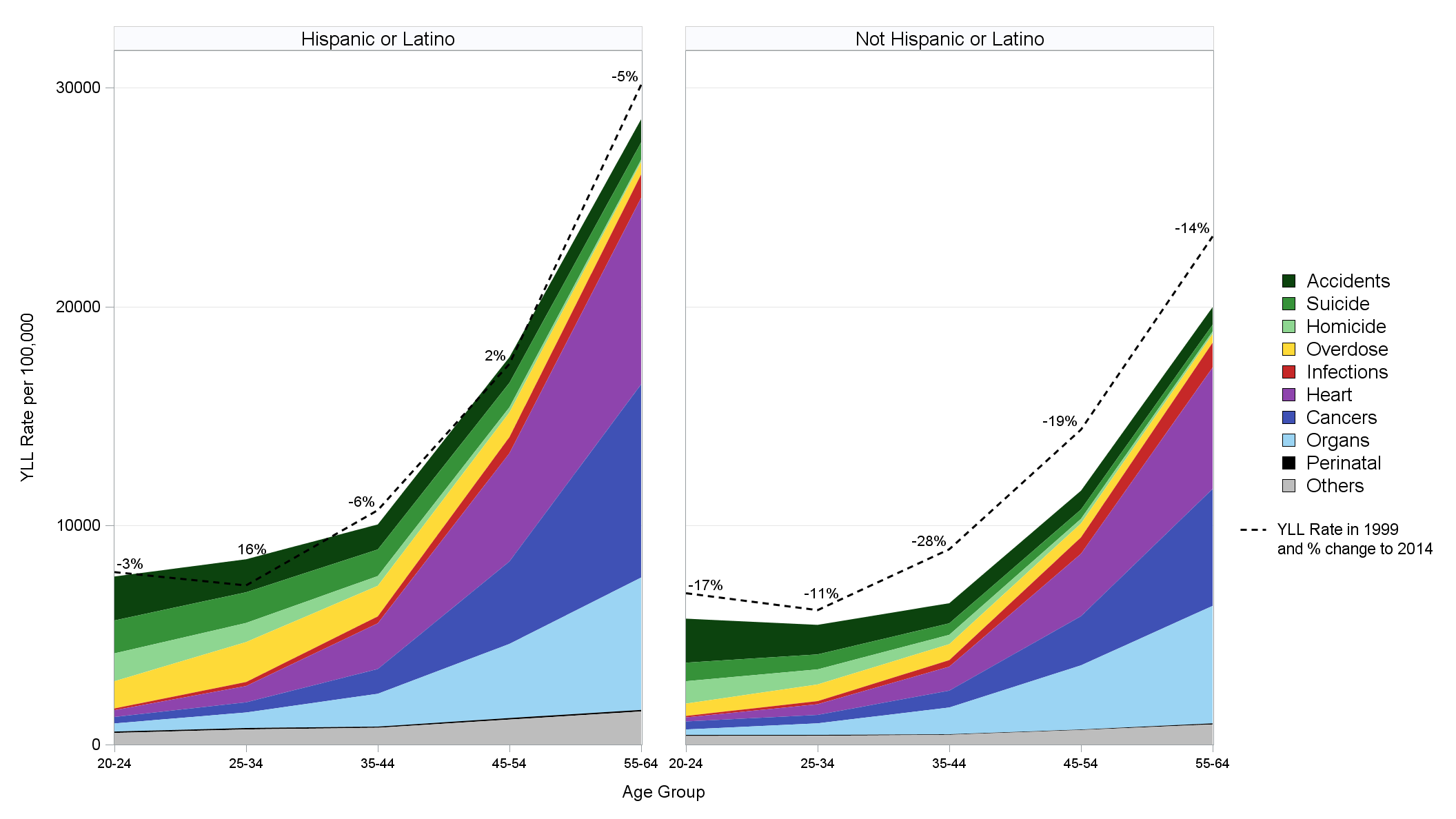


S5b Fig.


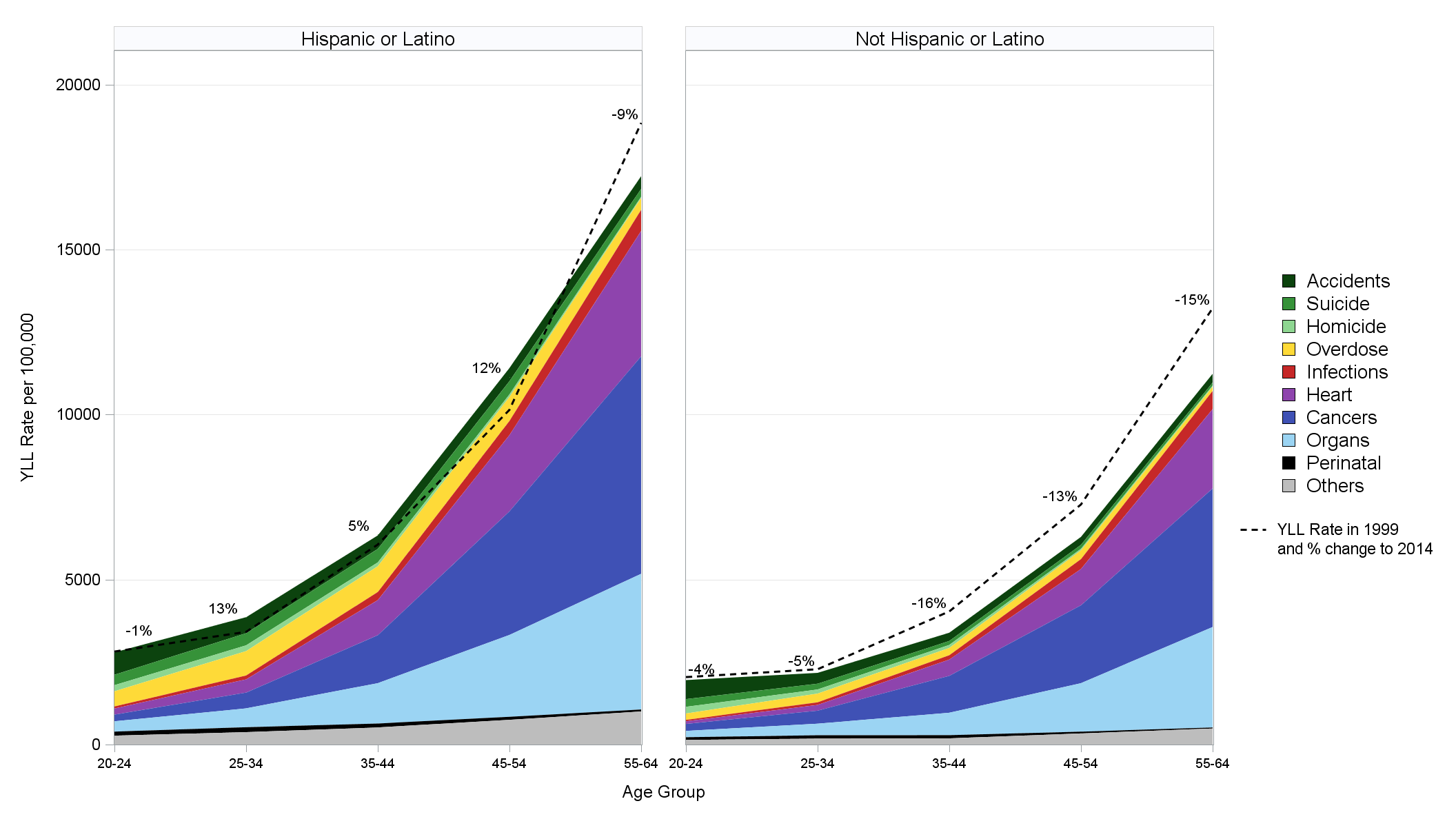

Supplement: S5 Fig — A. 2014 YLL by cause of death group for Hispanic and non-Hispanic males, ages 20–64, with comparison to and percent change from 1990 baseline. B. 2014 YLL by cause of death group for Hispanic and non-Hispanic females, ages 20–64, with comparison to and percent change from 1990 baseline. (DOCX) [file pone.0194308.s005.docx]
